# Point-of-care Ultrasound in the Diagnosis of Calciphylaxis

**DOI:** 10.5811/cpcem.2020.7.47886

**Published:** 2020-07-30

**Authors:** Natasha Tobarran, Mark Collin

**Affiliations:** Wellspan York Hospital, Department of Emergency Medicine, York, Pennsylvania

**Keywords:** Point-of-care ultrasound, calciphylaxis, necrotic skin ulcer

## Abstract

**Case Presentation:**

A 63-year-old male with a past medical history of end stage renal disease presented to the emergency department with painful, lower-extremity necrotic ulcerations. Ultrasound and computed tomography imaging showed concerns for calcium deposits. Biopsy confirmed the diagnosis of calciphylaxis, a rare lethal disease.

**Discussion:**

Emergency physicians should keep this disease on their differential due to the high mortality rate.

## CASE PRESENTATION

**Image 1:**
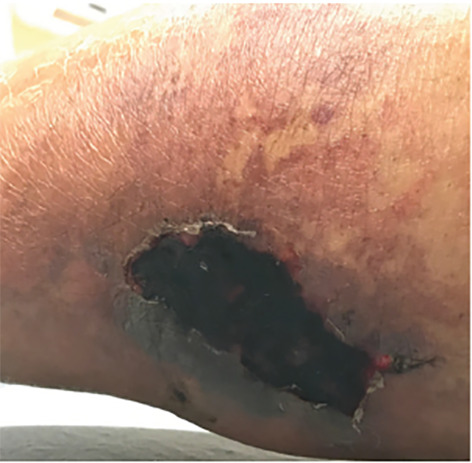
Physical examination revealing lower extremity skin necrosis due to calciphylaxis in the setting of end-stage renal disease.

**Image 2:**
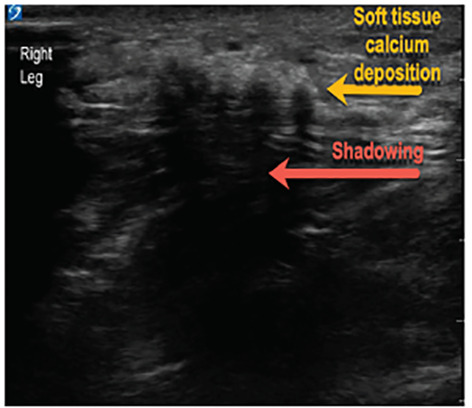
Soft tissue point-of-care ultrasound identifying soft tissue calcium deposits with shadowing diagnostic of calciphylaxis in a patient with end-stage renal disease.

**Image 3:**
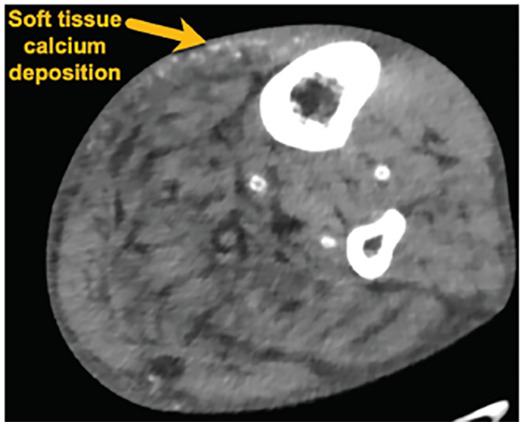
Computed tomography with axial view demonstrating soft tissue calcium deposits diagnostic of calciphylaxis in a patient with end-stage renal disease.

A 63-year-old male with end-stage renal disease (ESRD) presented to the emergency department with severe, bilateral lower-extremity pain with black necrotic ulcerations (Image 1). The symptoms began five weeks prior, and review of systems was negative for fevers or trauma. The patient was previously treated with antibiotics, prednisone, and oxycodone without improvement. Further evaluation via point-of-care ultrasound focusing on the necrotic areas revealed calcium deposits and shadowing (Image 2). Computed tomography confirmed soft tissue calcifications (Image 3).

## DISCUSSION

The findings were concerning for calciphylaxis. Punch biopsy showed extensive skin necrosis and calcifications confirming the diagnosis. The patient was treated with sodium thiosulfate and was discharged home but ultimately was transitioned to hospice care.

Calciphylaxis is rare and lethal disease, presenting with skin ischemia and necrosis caused by total occlusion of blood vessels secondary to calcification of arterioles and capillaries in the dermis and adipose tissue.^1^ The estimated six-month survival rate is 50%.^2^ It has been linked to ESRD, hyperparathyroidism, hypercalcemia, and hyperphosphatemia.^3^ Patients present with non-healing, painful necrotic skin lesions in areas with increased adiposity such as distal lower extremities.^2^ The diagnosis is clinical; however, biopsy can be used for confirmation. The treatment involves wound care, pain management, and correcting electrolyte abnormalities.^1^ Wound infection is a common complication. A trial of sodium thiosulfate, which blocks the calcification of vascular smooth muscle, may be implemented.^1^ It is important for emergency physicians to keep calciphylaxis on their differential for non-healing painful wounds, especially in high-risk patient populations. Point-of-care ultrasound is a useful tool in aiding with diagnosis.

**CPC-EM Capsule**

What do we already know about this clinical

Calciphylaxis is a rare disease with high morbidity and mortality presenting with painful necrotic lesions due to calcium deposits in the fat and skin.

What is the major impact of the image(s)?

Soft tissue calcium deposits with associated shadowing can be seen with ultrasound of the necrotic lesions, aiding in diagnosis of calciphylaxis.

How might this improve emergency medicine practice?

Point-of-care ultrasound may be useful for an astute clinician in the diagnosis of calciphylaxis, which should be considered when evaluating painful skin lesions.
